# Critical role of down-regulated in adenoma bicarbonate transporter in linaclotide stimulated intestinal bicarbonate secretion

**DOI:** 10.1101/2023.05.05.539132

**Published:** 2023-05-07

**Authors:** Jessica B. Sarthi, Annie M. Trumbull, Shayda M. Abazari, Vincent van Unen, Joshua E. Chan, Nam Soo Joo, Yanfen Jiang, Calvin J. Kuo, Zachary M. Sellers

**Affiliations:** 1 Department of Pediatrics, Division of Gastroenterology, Hepatology, and Nutrition, Stanford University, Palo Alto, CA, USA; 2 Department of Medicine, Division of Hematology, Stanford University, Palo Alto, CA, USA; 3 Department of Pediatrics, Division of Pulmonary, Asthma, and Sleep Medicine, Stanford University, Palo Alto, CA, USA

## Abstract

Duodenal bicarbonate secretion is critical to epithelial protection, nutrient digestion/absorption and is impaired in cystic fibrosis (CF). We examined if linaclotide, typically used to treat constipation, may also alter duodenal bicarbonate secretion. Bicarbonate secretion was measured in vivo and in vitro using mouse and human duodenum. Ion transporter localization was identified with confocal microscopy and de novo analysis of human duodenal single cell RNA sequencing (sc-RNAseq) was performed. Linaclotide increased bicarbonate secretion in mouse and human duodenum in the absence of CFTR expression or function. Linaclotide-stimulated bicarbonate secretion was eliminated by down-regulated in adenoma (DRA) inhibition, regardless of CFTR activity. Sc-RNAseq identified that 70% of villus cells expressed *SLC26A3*, but not *CFTR*, mRNA. Linaclotide increased apical membrane expression of DRA in non-CF and CF differentiated enteroids. These data provide insights into the action of linaclotide and suggest linaclotide may be a useful therapy for CF individuals with impaired bicarbonate secretion.

## INTRODUCTION

Proximal duodenal bicarbonate secretion, together with pancreatic bicarbonate secretion, is integral to preventing intestinal mucosal injury from gastric secretions and establishing a non-acidic intraluminal environment for activation of digestive enzymes. This importance is highlighted by cystic fibrosis (CF) intestinal disease and malnutrition, where decreased bicarbonate secretion is central to disease pathogenesis.^1,2^ Duodenal bicarbonate secretion is highly regulated, driven by luminal acid and neurohormonal signaling, ultimately resulting in coordinated transepithelial bicarbonate secretion by the cystic fibrosis transmembrane conductance regulator (CFTR), chloride/bicarbonate exchange (e.g., down-regulated in adenoma, DRA; putative anion transporter-1, PAT-1), and/or decreased proton transport through sodium/hydrogen exchange (e.g. NHE3).^3^ Despite its physiologic importance, there are limited therapies targeting duodenal bicarbonate secretion.

Linaclotide is a Food and Drug Administration (FDA)-approved medication for the treatment of constipation-type irritable bowel syndrome and chronic idiopathic constipation whose effect relies on its structural similarity to the heat-stable enterotoxin of *Escherichia coli* (STa). Both STa and linaclotide bind guanylyl cyclase C (GC-C) receptors on the apical surface of enterocytes and cause increases in CFTR-dependent chloride secretion and NHE3-dependent inhibition of sodium absorption.^4–6^ Previously, we identified that STa stimulates duodenal bicarbonate secretion through CFTR and CFTR-independent means.^7,8^ Linaclotide has been investigated as a potential therapy for CF-related constipation,^6,9,10^ however, it is unclear if linaclotide stimulates duodenal bicarbonate secretion and if linaclotide could improve impaired intraluminal pH in CF patients by stimulating CFTR-independent bicarbonate secretion.

We undertook a series of experiments utilizing in vivo and in vitro bicarbonate secretion measurements in mice and humans to examine the effect of linaclotide on duodenal bicarbonate secretion. We coupled these experiments with scRNA-seq analysis of acid-based transporters in the human duodenum and confocal microscopy to characterize the membrane expression of the DRA chloride/bicarbonate exchanger in human duodenum in the presence or absence of CFTR activity and/or expression.

## RESULTS

### Linaclotide stimulates bicarbonate secretion in mouse and human duodenum.

**Figure 1.**
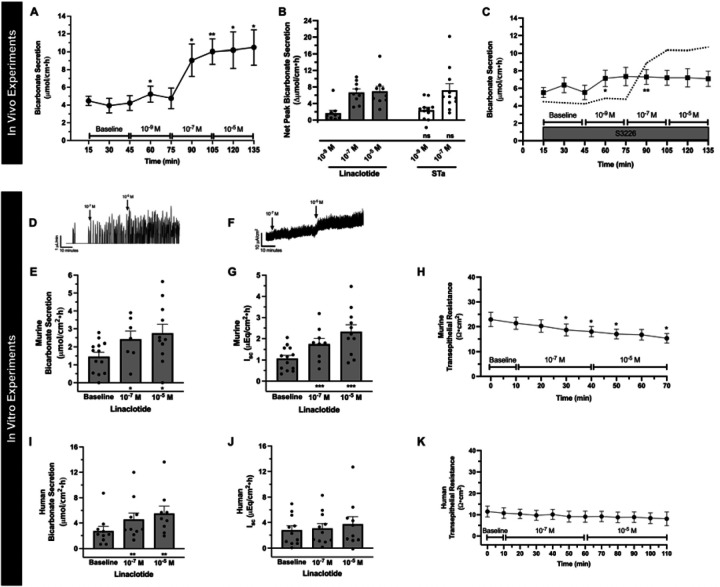
Linaclotide stimulates duodenal bicarbonate secretion in mice and humans. **A.** In vivo measurement of duodenal bicarbonate secretion in response to luminal perfusion of 10^−9^ M, 10^−7^ M, and 10^−5^ M linaclotide in C57Bl6/J adult mice (n=9). After 30-minute baseline measurements (saline perfusion), each dose was perfused for 30 minutes. Data are expressed as means ± SEM. *, P<0.05; **, P<0.01 vs. baseline by ANOVA. **B.** Net peak bicarbonate secretion measurements from in vivo experiments. Peak responses were subtracted from mean baseline values. For linaclotide, values were taken from experiments in (A). For STa, separate experiments were performed similar to (A), except with only 10^−9^ M and 10^−7^ M STa (luminal perfusion). Each point represents a different mouse. Columns indicate means ± SEM. *, P<0.05; **, P<0.01; ***, P<0.001 peak response vs. baseline by paired Student’s t-test; ns = no significant difference between STa and linaclotide by unpaired Student’s t-test. **C.** In vivo effect of linaclotide (10^−9^ M, 10^−7^ M, 10^−5^ M) in the presence of NHE3 inhibitor S3226 (10^−5^ M, luminal perfusion, n=10). Dotted line is the mean response to linaclotide (taken from Figure 1A). *, P<0.05; **, P<0.01 vs. baseline by ANOVA. **D.** Representative trace for in vitro bicarbonate secretion in mouse duodenum. Y-axis represents the amount of titrated HCl, which reflects amount of base secreted by the tissue. During baseline there are few peaks, but upon stimulation the number of titration events (and volume of titrant) increases, indicating increased bicarbonate secretion. **E.** Mean ± SEM in vitro mouse duodenal mucosal bicarbonate secretion during baseline and linaclotide stimulation (10^−7^ and 10^−5^ M, apical). Each point represents a separate piece of duodenum from total of 10 mice. *, P<0.05 vs. baseline by ANOVA. **F.** Representative short-circuit current (I_sc_) trace for in vitro mouse duodenum experiments. **G.** Mean ± SEM in vitro mouse duodenal I_sc_ during baseline and linaclotide stimulation (10^−7^ and 10^−5^ M, apical) after conversion of raw μA/cm^2^ data to μEq/cm^2^•h. Each point represents a separate piece of duodenum from the same 10 mice in (E). ***, P<0.001 vs. baseline by ANOVA. **H.** In vitro transepithelial resistance measurements from experiments in (E and G). *, P<0.05 vs. baseline by ANOVA. **I-K.** Human duodenal mucosal bicarbonate secretion (I), I_sc_ (J), and transepithelial resistance (K) measured from endoscopic biopsies. Experiments were performed and data expressed similar as murine experiments in Fig. 1D–H. **, P<0.01 vs. baseline by ANOVA.

To determine linaclotide’s ability to stimulate intestinal bicarbonate secretion, we examined its effect on the duodenal segment of the small intestine, given the robust expression of acid-base transporters in this segment that function to neutralize the highly acidic gastric contents it encounters. We first examined the effect of in vivo intestinal perfusion of linaclotide on duodenal bicarbonate secretion. Linaclotide stimulated robust and sustained increases in bicarbonate secretion at 10^−7^ M and 10^−5^ M (P<0.001 and P=0.004, n=9), with small and transient, increases in bicarbonate secretion over baseline secretory rates at 10^−9^ M (P=0.033, n=9). There was no significant difference between 10^−7^ M or 10^−5^ M (P=0.926) (Fig. 1a, b). These bicarbonate secretory rates were similar to those observed with STa (Figure 1b, 10^−9^ M: P=0.491 and 10^−7^ M: 0.792, respectively). Linaclotide did not stimulate net increases in luminal duodenal fluid (10^−9^ M: 0.01 ± 0.05, 10^−7^ M: −0.05 ± 0.06, 10^−5^ M: −0.04 ± 0.05 ∆mL/cm•h, P=0.856, 0.445, 0.367, respectively, n=8), similar to what we have previously reported for STa in the duodenum.^7^ As linaclotide can decrease proton secretion by inhibiting NHE3,^6,9^ we examined if linaclotide actively stimulates bicarbonate secretion, or purely inhibits baseline proton secretion to raise intraluminal pH. Using the same in vivo technique, perfusion with S3226 (10^−5^ M), a selective inhibitor of NHE3, caused a significant increase in baseline (3.78 ± 0.73 vs. 6.70 ± 0.68 μmol/cm•h, n=9–10, P=0.016). In the presence of S3226, linaclotide caused further increases in bicarbonate secretion (10^−9^ M: 2.22 ± 0.87, 10^−7^ M: 2.53 ± 0.82, 10^−5^ M: 2.38 ± 1.32 ∆μmol/cm•h, n=10, P=0.075, P=0.032, P=0.237, respectively)(Fig. 1c). In the presence of S3226, linaclotide-stimulated duodenal bicarbonate secretion was ~1/3 of the response without S3226 (10^−7^ M: 37.9 ± 12.3%, 10^−5^ M: 35.6 ± 19.8%). Thus, in mouse duodenum, linaclotide alters duodenal luminal pH through a combination of inhibiting NHE3-mediating proton secretion and stimulating bicarbonate secretion.

To further characterize linaclotide-stimulated bicarbonate secretion, we performed combined I_sc_ and bicarbonate secretory measurements in seromuscular-stripped mouse duodenum mounted in Ussing chambers. As seen in Fig. 1d–g, this in vitro method showed similar results as the in vivo method. Linaclotide stimulated significant increases in duodenal bicarbonate secretion at 10^−7^ M (P=0.025, n=9) and 10^−5^ M (P=0.047, n=11), with no difference between them (P=0.875). Short-circuit current analysis revealed that linaclotide stimulates significant increases in I_sc_ (10^−7^ M, P<0.001, n=9) and 10^−5^ M (P<0.001, n=11) (Fig. 1f, g). Bicarbonate secretion and I_sc_ were accompanied by small, but significant (P<0.05) changes in transepithelial resistance (Fig. 1h).

We next examined the potential for linaclotide to stimulate bicarbonate secretion in human duodenum using endoscopically obtained duodenal biopsies from subjects without known acid-base disturbances or histologic evidence of duodenal disease. Biopsies were mounted in Ussing chambers and combined bicarbonate secretion and I_sc_ measurements were performed, similar to Fig.1d–h. As seen in Fig. 1i, 10^−7^ M and 10^−5^ M linaclotide stimulated significant increases in human duodenal bicarbonate secretion (P=0.006, n=11 and P=0.004, n=10, respectively), at similar magnitudes previously reported for STa in human duodenal biopsies.^11^ In contrast to mice, linaclotide did not stimulate a significant change in I_sc_ in human biopsies (10^−7^ M: P=0.071, n=11; 10^−5^ M: P=0.186, n=10) (Fig. 1j) or transepithelial resistance (Fig. 1j, k). Thus, linaclotide stimulates electrogenic bicarbonate secretion in mouse duodenum and electroneutral bicarbonate secretion in human duodenum.

### Linaclotide-stimulated duodenal bicarbonate secretion occurs independent of CFTR expression or function.

**Figure 2.**
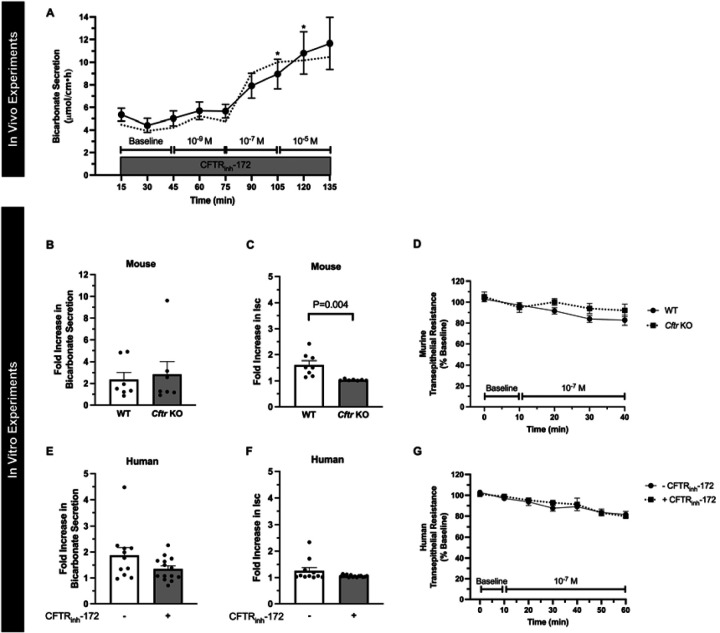
Linaclotide stimulates duodenal bicarbonate secretion independent of CFTR. **A.** In vivo measurement of duodenal bicarbonate secretion in mice, similar to Figure 1A, with the exception that each perfusate also contained CFTR_inh_-172 (2 × 10^−5^ M). Circles and whiskers represent means ± SEM (n=12). Dotted line is the mean response in the absence of CFTR_inh_-172 (taken from Figure 1A). *, P<0.05 vs. baseline by ANOVA. **B and C.** In vitro duodenal mucosal bicarbonate secretion (B) and I_sc_ (C) in wildtype and *Cftr* KO mice. Data are expressed as fold increase in linaclotide (10^−7^ M) stimulated responses over baseline bicarbonate secretion or I_sc_ in the same mouse. Columns with whiskers represent mean ± SEM with each dot representing a different experiment. Lack of P values indicates no significant difference. **D.** Transepithelial resistance measurements in wildtype (WT) and *Cftr* KO mice. **E-G.** In vitro duodenal mucosal bicarbonate secretion (E), I_sc_ (F), and transepithelial resistance (G) in human endoscopic biopsies with or without CFTR_inh_-172 pre-treatment (2 × 10^−5^ M, 40–60 minutes). Columns with whiskers represent mean ± SEM with each dot representing a different experiment. Lack of P values indicates no significant difference.

Next, given our prior findings that STa can stimulate CFTR-independent duodenal bicarbonate secretion in mice,^7^ we studied linaclotide’s dependence on CFTR for bicarbonate secretion. We first repeated the in vivo bicarbonate secretion experiments by perfusing the duodenum with CFTR_inh_-172, a specific CFTR inhibitor.^12^ Linaclotide continued to stimulate significant bicarbonate secretory responses at 10^−7^ M and 10^−5^ M (P=0.008 and P=0.018, respectively, n=12), but not 10^−9^ M (P=0.198, n=12), in the presence of CFTR_inh_-172 (2 × 10^−5^ M). In comparison, in vivo, CFTR_inh_-172 inhibited forskolin- stimulated (10^−4^ M) bicarbonate secretion by 65.1 ± 12.5%, preventing significant increases over baseline (P=0.778, n=6–9) (Supplementary Fig.1). In vitro, sequential addition of CFTR inhibitors with different mechanisms of action, GlyH-101 (10^−5^ M, mucosal) and glibenclamide (3 × 10^−4^ M, mucosal), following CFTR_inh_-172 (2 × 10^−5^ M, serosal), resulted in no further decrease in percent inhibition of forskolin-stimulated I_sc_ (0.2 ± 0.3% and −0.9 ± 1.3%, respectively, n=2, P=0.581). To expand upon these pharmacologic experiments, we performed in vitro Ussing chamber experiments with CFTR^tm1UNC^ knockout (KO) mice, which have global KO of the *Cftr* gene.^13^ Given the similar responses between 10^−7^ M and 10^−5^ M in prior experiments, for these studies we specifically focused on 10^−7^ M linaclotide. We found that linaclotide (10^−7^ M) stimulated significant increases in duodenal bicarbonate secretion in *Cftr* KO mice (P=0.028, n=7) that were similar to wildtype (WT) mice (P=0.728) (Fig. 2b). In contrast to mice expressing CFTR, linaclotide stimulated minimal change in I_sc_ in *Cftr* KO mice (0.77 ± 0.31 ∆μA/cm^2^, P=0.049, n=8) with no change in transepithelial resistance (P=0.396) (Fig. 2c, d), suggesting that in the absence of CFTR, linaclotide stimulates electroneutral bicarbonate secretion. To determine if these findings hold true in humans, we pre-treated human endoscopic duodenal biopsies with CFTR_inh_-172 and then performed repeat Ussing chamber measurements. Linaclotide (10^−7^ M) continued to stimulate significant increases in duodenal bicarbonate secretion in biopsies pre-treated with CFTR_inh_-172 (P=0.024, n=14), at a magnitude that was comparable to biopsies without CFTR_inh_-172 treatment (P=0.644) (Fig. 2e). There was no change in linaclotide-stimulated I_sc_ with or without CFTR_inh_-172 treatment (0.27 ± 0.11 vs. 0.13 ± 0.04 ∆μEq/cm^2^•h, P=0.239, n=11–14) (Fig. 2f). Transepithelial resistance in the presence of CFTR_inh_-172 was unchanged before or after linaclotide stimulation (P=0.889, n=14) (Fig. 2g).

### Linaclotide-stimulated duodenal bicarbonate secretion is mediated by the DRA chloride/bicarbonate exchanger.

**Figure 3.**
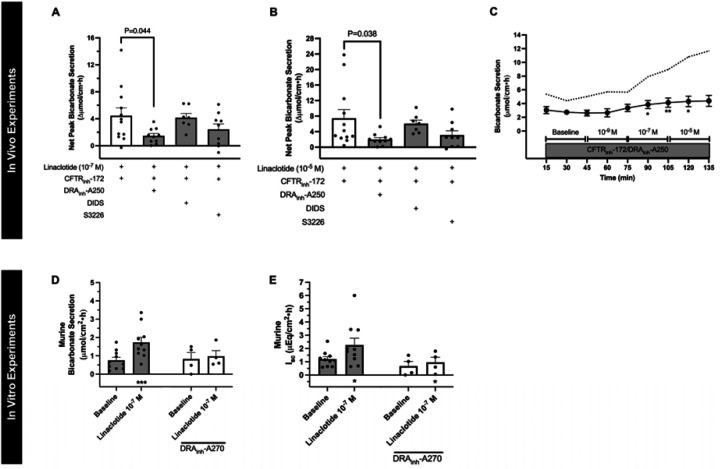
Critical role of DRA in linaclotide-stimulated duodenal bicarbonate secretion. **A and B.** To determine the source of linaclotide-stimulated bicarbonate transport in the absence of CFTR function, in vivo experiments were repeated, similar to Figure 2A, except in addition to CFTR_inh_-172 (2 × 10^−5^ M), one of the following was also added to the luminal perfusate: DRA_inh_-A250 (10^−5^ M), DIDS (2 × 10^−4^ M), or S3226 (10^−5^ M). Columns with whiskers are means ± SEM with each dot representing a different mouse. Significance was determined by ANOVA. **C.** Time course with linaclotide dose response in the presence of CFTR_inh_-172 (2 × 10^−5^ M) and DRA_inh_-A250 (10^−5^ M), as indicated by circles and whiskers. Data are mean ± SEM. Dotted line indicates mean response in the presence of CFTR_inh_-172 (2 × 10^−5^ M) only, as indicated in Figure 2. *, P<0.05; **, P<0.01 vs. baseline by ANOVA. **D.** Mean ± SEM in vitro mouse duodenal mucosal bicarbonate secretion (D) and I_sc_ (E) during baseline and linaclotide stimulation (10^−7^ M, apical) in the presence (grey columns) or absence (white columns) of DRA_inh_-A270 (10^−5^ M, bilateral). Each point represents a separate piece of duodenum from total of 2–4 mice. *, P<0.05; ***, P<0.001 vs. baseline by paired Student’s t-test.

To investigate the potential mechanism whereby linaclotide increases duodenal bicarbonate secretion independent of CFTR, we investigated the effect of DRA_inh_-A250 (selective inhibitor of DRA), DIDS (DRA-insensitive, non-selective anion exchanger inhibitor), and S3226 (selective inhibitor of NHE3) on duodenal bicarbonate secretion. Using our in vivo model, in separate experiments, each of these drugs were combined with CFTR_inh_-172 and perfused prior to stimulation with linaclotide. DRA_inh_-A250 (10^−5^ M) caused a 67 ± 8% reduction in CFTR-independent linaclotide-stimulated duodenal bicarbonate secretion at 10^−7^ M linaclotide (P=0.044, n=10), and 73 ± 6% inhibition at 10^−5^ M linaclotide (P=0.038, n=10) (Fig. 3a–c). In contrast, DIDS (2 × 10^−4^ M) failed to significantly impact CFTR-independent linaclotide-stimulated duodenal bicarbonate secretion (10^−7^ M: −7 ± 14%, P=0.993, n=7; 10^−5^ M: −19 ± 14%, P=0.893, n=7). S3226 (10^−5^ M) reduced CFTR-independent linaclotide-stimulated duodenal bicarbonate secretion by 45 ± 19% (10^−7^ M) and 58 ± 16% (10^−5^ M); however, neither of these were statistically significant (P=0.254, P=0.136, respectively, n=9) (Fig. 3a, b). Therefore, it appears that DRA is the primary source of bicarbonate transport for CFTR-independent linaclotide-stimulated duodenal bicarbonate secretion.

To determine if DRA’s critical role in linaclotide-stimulated duodenal bicarbonate secretion is unique to when there is functional loss of CFTR, we examined the role of DRA in mouse duodenal bicarbonate secretion measured in vitro. For these experiments, DRA_inh_-A270 was used, which has similar DRA specificity and inhibitory properties as DRA_inh_-A250 at 10^−5^ M.^14^ As seen in Fig. 3d, DRA_inh_-A270 (10^−5^ M) prevented linaclotide-stimulated duodenal bicarbonate secretion (10^−7^ M, P=0.524, n=4–10) with 86.3± 19.0% reduction from linaclotide alone.

### DRA is highly expressed in human duodenum.

**Figure 4.**
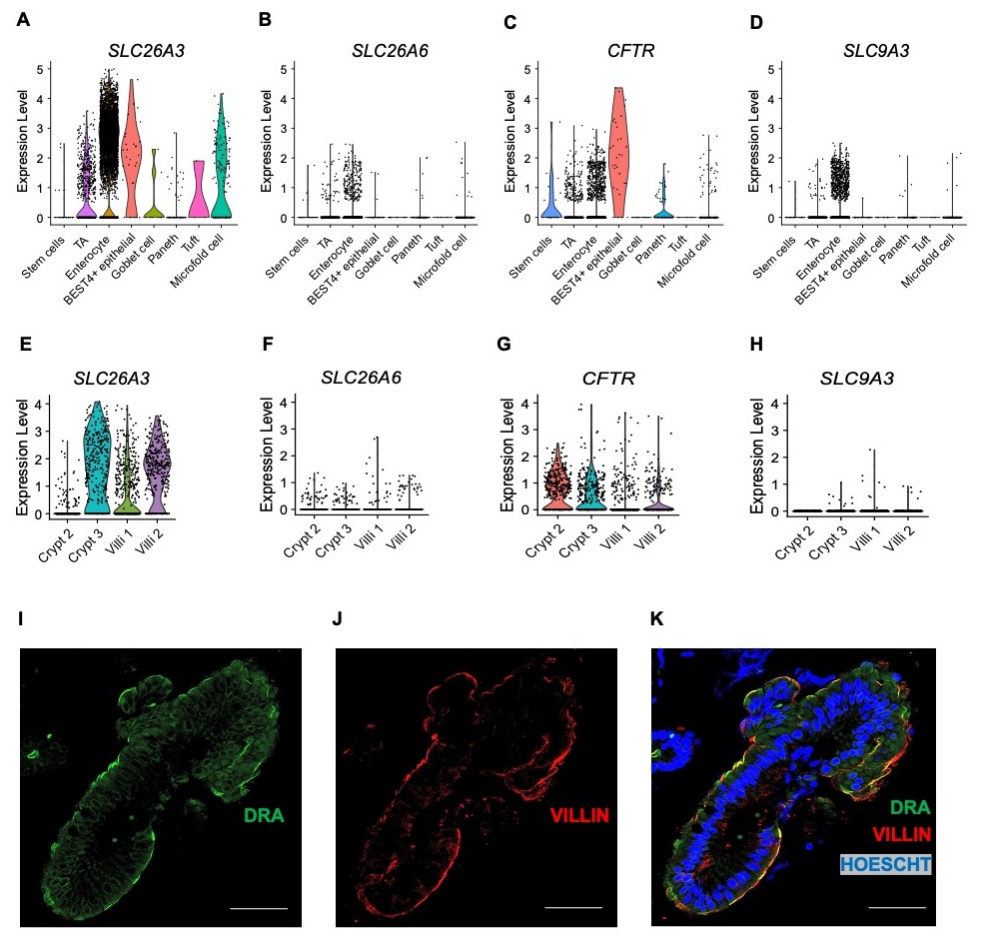
Cellular and membrane expression of *SLC26A3* (DRA) in human duodenal enterocytes. **A-H.** Cellular mRNA expression of *SLC26A3* (DRA), *SLC26A6* (PAT-1), *CFTR*, and *SLC9A3* (NHE3) based on re-analysis of Elmentaite et al.^19^ (A-D) and Busslinger et al.^18^ (E-H). Violin plots represent expression relative to all cells of that type, with each dot representing the expression of individual cells within each type. **I-K.** Confocal immunofluorescence imaging of DRA (I), villin (marker of apical membrane, J), and nucleus (K) in human duodenum from endoscopic biopsy. Scale bar = 20 μm.

DRA and PAT-1 are the primary chloride/bicarbonate exchangers expressed in mouse intestine, with PAT-1 showing dominant RNA expression in the small intestine and DRA RNA being more abundant in the colon.^15^ There is limited data regarding DRA expression in human duodenum.^16,17^ We capitalized on two previously published human duodenum scRNA-seq datasets^18,19^ to examine DRA expression in human duodenum. The Elmentaite et al. dataset^19^ contained 5,944 cells with an average of 4,164 reads per cell from 5 healthy adult duodenal samples, allowing for high confidence in cell-type separation. The Busslinger et al. dataset^18^ contained 702 crypt cells and 923 villus cells from 2 adult duodenal samples, with an average of 10,381 reads per cell. Data from one crypt sample (“Crypt 1”) was excluded due to very low sequence reads (2,088 per cell). We verified accurate classification into crypt and villus cells by examining *LGR5* expression and proliferation markers *MKI67* and *PCNA*, which are generally restricted to the crypt region. These were all more abundant in the crypt group, with *LGR5* being exclusively expressed in the crypt, but not villus, group (Supplementary Fig. 2a–c). Using these two datasets, we examined expression of the key acid-base transporters in the duodenum, DRA (*SLC26A3*), PAT-1 (*SLC26A6*), *CFTR*, and NHE3 (*SLC9A3*). Our analysis showed that *SLC26A3* mRNA is predominantly expressed in enterocytes (Fig. 4a), in both the crypt and villus groups (Fig. 4e), with higher expression and expressed in more cells than *SLC26A6*, *CFTR*, or *SLC9A3* (Fig. 4a–d and fe–h). We also found high protein expression in the duodenum when performing immunofluorescence against DRA in human duodenal biopsies (n=3). At baseline, the majority of DRA is localized to the intracellular apical region, with a smaller fraction present at the apical membrane (Fig. 4i–k).

**Figure 5.**
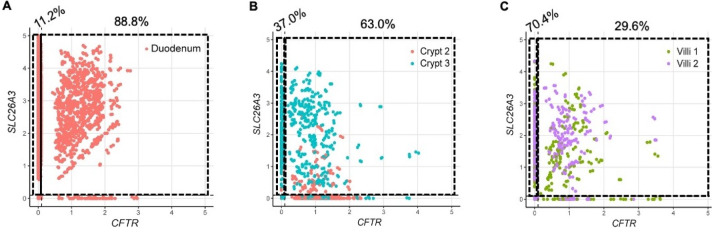
*SLC26A3* and *CFTR* co-expression in human duodenum by single cell RNA sequencing. **A-C.** Co-expression of *SLC26A3* (DRA) and *CFTR* mRNA using FeatureScatter based on Elmentaite et al.^19^ enterocytes (A) and Busslinger et al.^18^ crypt and villi (B and C) datasets that were analyzed in Figure 4. Numbers on top of graphs represent the percentage of *SLC26A3*-expressing cells that express *SLC26A3* only (left) or *SLC26A3* and *CFTR* (right).

Given DRA’s functional independence from CFTR in mediating linaclotide-stimulated duodenal bicarbonate secretion, we quantified the proportion of *SLC26A3*-expressing cells that co-expressed *CFTR*. In the Elmentaite et al. dataset, 88.8% of *SLC26A3*-expressing enterocytes co-expressed *SLC26A3* and *CFTR* (Fig. 5a). Using the Busslinger et al. dataset to examine crypt vs. villus localization, 63.0% of *SLC26A3* positive crypt cells co-expressed *SLC26A3* and *CFTR*, whereas in the villus cells, only 29.6% did so (Fig. 5b, c). These findings were not ubiquitous for all chloride/bicarbonate exchangers. *SLC26A6* and *CFTR* co-expression was relatively low in enterocytes (16.7%) (Supplementary Fig. 3a), but examination of the crypt and villus cell types showed a higher percentage of *CFTR* co-expression with *SLC26A6* than *SLC26A3*, with 81.8% of crypt cells and 42.9% of villus cells co-expressing *SLC26A6* and *CFTR* (Supplementary Fig. 3b, c). In the Elmentaite et al. dataset, *SLC9A3* and *CFTR* were co-expressed in 14.3% of enterocytes, similar to *SLC26A6* (Supplementary Fig. 3d). The Busslinger et al. dataset possessed insufficient numbers of *SLC9A3*-expressing cells to analyze *SLC9A3* and *CFTR* co-expression in crypts and villi.

### Linaclotide alters the apical localization of DRA.

**Figure 6.**
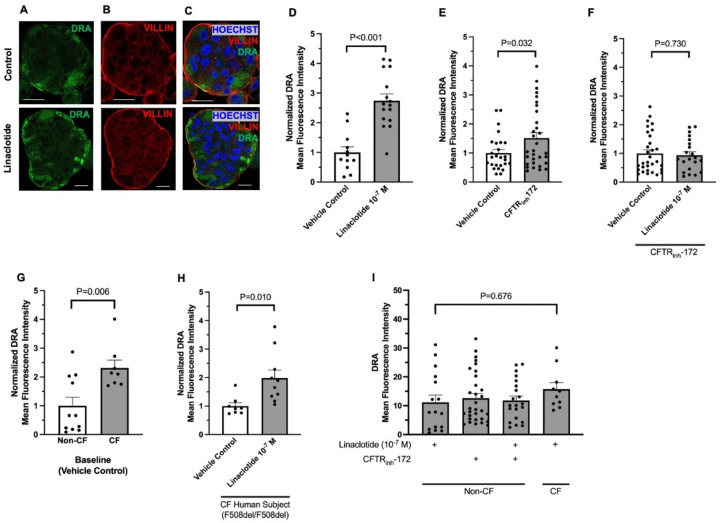
Linaclotide increases membrane expression of DRA in apical-out human duodenal enteroids. **A.** Representative confocal microscopy images of DRA (A), villin (B), and DRA and villin and Hoescht (C) during control conditions (water, 40 minutes, top) or linaclotide (10^−7^ M, 40 minutes, bottom). **D-F.** Quantification of DRA present at the apical membrane using villin to define apical membrane. Apical membrane DRA mean fluorescence intensity following CFTR_inh_-172 (2 × 10^−5^ M, 40 minutes) and/or linaclotide (10^−7^ M, 40 minutes) treatment was normalized to vehicle controls for comparison. Vehicle control for linaclotide was water and for CFTR_inh_-172 was DMSO. Columns with whiskers are mean ± SEM with each dot representing a different enteroid. Enteroids from three different patients were used for each condition. Significance determined by unpaired Student’s t-test. For enteroids treated with both CFTR_inh_-172 and linaclotide, enteroids were pretreated with CFTR_inh_-172 for 40 minutes prior to linaclotide treatment. **G and H.** DRA membrane expression in non-CF and CF (F508del homozygous) enteroids during vehicle control conditions (G, water) or during linaclotide stimulation (H, 10^−7^ M, 40 minutes). N=7–11 enteroids from 1 patient across 2 different passages. Data were normalized to non-CF enteroids (G) or vehicle control in CF enteroids (H). Significance determined by unpaired Student’s t-test. **I.** To compare DRA membrane expression across non-CF and CF enteroids in different situations, absolute DRA membrane mean fluorescence intensity was plotted. Data are from experiments performed in D-H. Significance determined by ANOVA.

Ion channel activity can be regulated by altering membrane expression of channels. We examined if linaclotide alters DRA activity by changing its apical membrane expression. For these experiments we utilized human derived three-dimensional duodenal enteroids. Given that the linaclotide receptor GC-C is expressed at the apical surface, we generated reverse polarity apical-out duodenal enteroids, which allow facile access to the apical membrane via the enteroid exterior (Supplementary Fig. 4a, b).^20^ Using qPCR, we verified that apical-out enteroids express *CFTR*, *SLC26A3, SLC26A6, SLC9A3, and GUCY2C* in undifferentiated and 3-day differentiated enteroids (Supplementary Fig. 4c, d). In contrast, *LGR5* was decreased in differentiated apical-out enteroids. Using this model of human duodenum, we incubated apical-out differentiated enteroids with linaclotide (10^−7^ M, 40 minutes) or vehicle control (water, 40 minutes) and performed confocal immunofluorescence to localize DRA. As seen in Figures 6a–d, linaclotide significantly increased apical membrane expression of DRA. In contrast, when we performed the same experiment with NHE3 localization, we found no change in the apical expression of NHE3 upon linaclotide stimulation (Supplementary Fig. 5a–d). We verified that myosin VI (*MYO6*), a key protein in NHE3 trafficking,^21^ mRNA was present in apical-out enteroids. *MYO6* was expressed at similar levels in this model as many of the other transporters we examined (Supplementary Fig. 4c, d). To determine if linaclotide also stimulates DRA localization upon lack of functional CFTR, we repeated the experiments in the presence of CFTR_inh_-172 (2 × 10^−5^ M, 40 minutes). Compared to vehicle control (DMSO, 40 minutes), CFTR inhibition resulted in an increase in the apical expression of DRA (P =0.032, n=26–31)(Fig. 6e), but not NHE3 (P=0.633, n=16–30) (Supplementary Fig. 5e). Of note, DMSO alone increased DRA membrane expression compared to water (MFI means ± SEM: 10.94 ± 1.32 vs. 3.43 ± 1.03, n=11–15, P<0.001). Linaclotide stimulation in the presence of CFTR_inh_-172 did not alter DRA or NHE3 expression (P= 0.730, n=31–31; P=0.535, n=29–30, respectively) (Fig. 6f, Supplementary Fig. 5f).

To determine how our CFTR_inh_-172 studies compare to chronic loss of CFTR expression and function, we examined DRA membrane expression in human duodenal enteroids isolated from a CF subject with F508del homozygous CFTR mutations. Comparison of baseline (water vehicle control, 40 minutes) DRA membrane expression between non-CF and CF enteroids showed CF enteroids had increased DRA expression at the apical membrane (Fig. 6g, n=10–11, P=0.006). Linaclotide stimulation (10^−7^ M, 40 minutes) increased DRA membrane expression in F508del homozygous CF enteroids, compared to CF enteroids treated with vehicle control (water, 40 minutes)(Fig. 6h, n=8–10, P=0.010). To examine the discrepancies between CFTR_inh_-172 + linaclotide in non-CF enteroids and linaclotide in the CF enteroids, we examined the absolute mean fluorescent intensities (MFI) of each condition. We found that CFTR_inh_-172 alone caused similar increases in absolute DRA membrane expression as linaclotide in non-CF enteroids (with or without CFTR_inh_-172) or CF enteroids (Fig. 6i, n=10–31, P=0.676), suggesting that lack of additional DRA membrane expression in non-CF enteroids treated with CFTR_inh_-172 + linaclotide may be due to maximal DRA membrane expression already being achieved upon CFTR_inh_-172 treatment. Taken together with our bicarbonate secretory measurements, these data show that linaclotide can alter DRA activity and/or membrane expression, in the presence or absence of functional CFTR. In contrast, linaclotide appears to modulate NHE3 activity, rather than membrane expression, regardless of CFTR function.

## DISCUSSION

Numerous studies in animal models and humans have shown the critical importance of duodenal bicarbonate secretion in maintaining epithelial integrity and creating the appropriate intestinal intraluminal pH for digestion. In CF, with genetic loss of CFTR, or *Helicobacter pylori* infection, with down-regulation of CFTR and PAT-1,^22^ decreased duodenal mucosal bicarbonate secretion contributes to malabsorption and duodenal ulcers, respectively. While eradication of *Helicobacter pylori* with antibiotics may restore bicarbonate function, the most common way to improve small intestinal pH in CF is through chronic gastric acid suppression. Ivacaftor, a CFTR potentiator, was shown to improve proximal intestinal pH in CF patients with the G551D CFTR gating mutation (accounts for 4–5% of CFTR mutations).^23^ While additional CFTR modulators are FDA-approved, it remains unknown whether these positively impact intestinal pH. This gap, coupled with lack of potential bicarbonate-correcting therapy for CF patients ineligible for CFTR modulator therapies (due to genotype or medical contraindication), has driven us to seek ways to improve intestinal bicarbonate secretion independent of CFTR. Our current data are in line with prior mouse and human duodenum studies with STa^7,11^ and a recent study by Tan et el. showing that linaclotide can stimulate jejunal bicarbonate secretion in mice.^9^ Uniquely, we have shown that linaclotide stimulates both mouse and human bicarbonate secretion in the duodenum, where bicarbonate secretion is physiologically important. Furthermore, we have provided evidence for the roles (or lack of) membrane trafficking of DRA and NHE3 in this process.

Linaclotide represents an attractive target for restoration of deficient duodenal bicarbonate secretion in CF. Our data in both mice and humans provide greater confidence in the translatability of our results to clinical care. While we did not study other segments of the small intestine, linaclotide stimulates jejunal bicarbonate secretion in F508del CF mice.^9^ Braga Emidio et al. identified that linaclotide’s half-life in small intestinal fluid was 48 minutes,^24^ suggesting that its effect is likely to be most influential in the proximal small intestine. Comparing linaclotide-stimulated and acid-stimulated duodenal bicarbonate secretion using the same methodology,^25^ the linaclotide effect is about 75% of the stimulatory effect of acid, indicating linaclotide could produce a clinically meaningful impact for patients. With existing FDA approval, and the use of clinically relevant dosing,^9^ our results may provide immediate application to patients.

In the absence of CFTR, the logical targets for intestinal pH modulation are either 1) increasing chloride/bicarbonate exchanger activity, and/or 2) decreasing sodium/hydrogen exchange. Linaclotide increases intraluminal small intestinal fluid in mice by inhibiting NHE3-dependent sodium absorption.^6,9^ Another byproduct of NHE3 inhibition is a decrease in proton secretion, which can raise intraluminal pH. Consistent with this, NHE3 inhibition in our studies “increased bicarbonate secretion,” likely by producing less luminal protons to neutralize secreted bicarbonate anions. In our NHE3 trafficking studies, we did not see a significant decrease in NHE3 membrane localization with linaclotide treatment. NHE3 activity may be impacted by shifting along the microvilli,^21^ however, our imaging resolution was unable to determine these potential nanometer shifts. Nonetheless, our in vivo data supports that NHE3 inhibition alone does not account for linaclotide’s ability to increase duodenal pH. Our studies using specific DRA inhibitors and DRA protein trafficking studies in apical-out human enteroids, indicate that DRA-mediated chloride/bicarbonate exchange is critical to linaclotide stimulated duodenal bicarbonate secretion. This helps explain the lack of linaclotide-stimulated short-circuit current in *Cftr* KO mice, human biopsies with CFTR_inh-_172, and prior studies by McHugh et al. in F508del mice.^6^ Interestingly, we found that CFTR inhibition alone caused an increase in DRA membrane trafficking. There was also increased apical membrane DRA expression in F508del homozygous duodenal enteroids even without stimulation. In mouse intestinal crypts, Strubberg et al. identified that intracellular pH was raised in *cftr* KO mice or wildtype mice treated with CFTR_inh_-172.^26^ In fact, ~20 years ago, Kaunitz et al. proposed the “CF Paradox” whereby diminished bicarbonate transport may increase intracellular buffering in CF and prevent duodenal ulcer formation despite diminished bicarbonate secretion.^27^ Thus, we hypothesize that upon CFTR blockade (or genetic lack of *CFTR*), the rise in intracellular pH may trigger an increase in DRA expression and protein localization to at least partially restore bicarbonate secretion and limit the intracellular pH rise. Bijvelds et al. recently reported a nearly 2,000-fold increase in *DRA* mRNA in a CF ileal biopsy compared to non-CF ileum (n=1 each).^28^ The lack of additional membrane trafficking of DRA in the presence of CFTR inhibition may indicate that DRA trafficking requires CFTR function or that a ceiling affect for DRA membrane expression had already been achieved. Based on our current data we favor the latter interpretation since linaclotide increased DRA trafficking in F508del homozygous CF enteroids and a comparison of MFIs across conditions showed that while the relative magnitude of DRA trafficking may differ between vehicle controls/conditions, the absolute magnitudes are similar. Further study on how DRA and other acid-base transporters are altered in CF intestine, especially in human samples, may identify new potential therapies for CF intestinal disease.

Prior to our study, our knowledge on the intestinal expression profile of DRA and other acid-base transporters was largely based on mouse studies. Wang et al. reported high expression of *Slc26a3* (DRA) RNA in the colon, but negligible RNA expression in the small intestine (duodenum, jejunum, ileum). Conversely, *Slc26a6* (PAT-1) RNA expression was high in mouse duodenum, jejunum, and ileum, with minimal in the colon.^15^ Yet, *Slc26a3* KO mouse studies suggest a prominent role for DRA in basal and stimulated bicarbonate transport. ^29,30^ Human studies are sparse. Yin et al. used human duodenal enteroids to examine *SLC26A3*, *SLC26A6*, and *SLC9A3* (NHE3) mRNA expression in undifferentiated and terminally differentiated human duodenal enteroids upon differentiation.^17^ Taking advantage of newly published scRNA-seq datasets from the human duodenum, we have provided a comparison on the expression of *SLC26A3*, *SLC26A6*, and *SLC9A3*, from human duodenum tissue. *SLC26A3* mRNA was generally expressed ~3–4x higher than *SLC26A6*, *CFTR*, and *SLC9A3*. In the CFTR high expressing BEST4+ cells, *CFTR* expression levels approached that of *SLC26A3*. Of note, *SLC26A3* was also highly expressed in BEST4+ cells, although enterocytes comprise the majority of *SLC26A3*-expressing cells. These findings, put in context of prior studies, suggest a more prominent role for DRA in the human duodenum than may have generally been appreciated from mouse studies alone. Future work examining not only the mRNA expression, but also protein expression, and especially pertinent to membrane ion transporters, membrane localization of DRA, PAT-1, and NHE3 across different segments of human intestine should advance our knowledge regarding the regional roles of these acid-base transporters in health and disease.

In summary, we have identified that linaclotide may have more pleiotropic ion transport effects beyond CFTR- and NHE3-mediated chloride and sodium transport, respectively. We have shown, using mice, duodenal biopsies, and apical-out human duodenal enteroids, that linaclotide stimulates bicarbonate secretion, and does so at least in part, through DRA recruitment to the apical membrane. Our findings that this remains possible independent of CFTR activity and/or expression has potentially important implications for CF patients, who may benefit from linaclotide’s bicarbonate stimulatory effect, even if they can’t leverage its pro-chloride secretory properties.^6^ Furthermore, our identification of high DRA expression in the human duodenum may spur interest in identifying DRA-activating drugs targeting proximal small intestinal disease.

## METHODS

### Chemicals.

CFTR_inh_-172 was purchased from Cayman Chemical (Ann Arbor, MI), 4,4’-Diisothiocyanatostilbene-2,2’-disulfonate (DIDS) and S3226 were purchased from Sigma-Aldrich (St. Louis, MO). DRA_inh_-A250 and DRA_inh_-A270 were gifts from Drs. Alan Verkman and Onur Cil at the University of California, San Francisco. Linaclotide was purchased from Santa Cruz Biotechnology, Inc (Dallas, TX).

### In Vivo Measurement of Bicarbonate Secretion.

In vivo measurement of duodenal bicarbonate secretion was performed using a well-validated technique.^31^ Animals were maintained with free access to food and water for up to 1 hour before the experiment, then fasted from chow. Mice were anesthetized with oxygen-delivered isoflurane (1–3%) at 1 L/min via a vaporizer (Braintree Scientific, Inc, Braintree, Mass). Mouse temperature was monitored by rectal probe and maintained at 37°C through automated warming using a controlled warming pad (ATCC 2000, World Precision Instruments, Sarasota, FL). Anesthetic plane was assessed by respiratory rate and toe pinch reflex. Respiratory and heart rates were determined every 15 min. Animals could be sustained for more than 3 hours under these experimental conditions.

To isolate the proximal duodenum, the abdomen was opened by a central vertical incision, and the proximal 5–10 mm of duodenum, from the pylorus to just proximal to the entry of the common bile duct, was isolated in situ without compromising vascular supply. A small polyethylene tube (PE-50) with a distal flange was advanced to the duodenal bulb via the stomach, and a ligature was secured around the pylorus. A distal intestinal incision was made, and PE-50 flanged tubing was advanced to just proximal to the entry of the pancreaticobiliary duct to prevent entry of the pancreatic or biliary secretions and allow for collection of effluent from isolated segment. The isolated duodenal segment was gently flushed and then continuously perfused (Harvard Infusion Pump, Harvard Apparatus, South Natick, MA, USA) at a rate of 0.21 mL/min with 154 mM NaCl (37°C) ± stimulatory/inhibitor drugs. Effluents from the isolated segment were visually free of bile and blood throughout all experiments. After an initial 15-minute washout period, basal bicarbonate secretion was measured for 30 minutes. Subsequently, linaclotide (10^−9^ M, 10^−7^ M, 10^−5^ M) ± DRA_inh_-A250 inhibitor (10^−5^ M), CFTR_inh_-172 (2 × 10^−5^ M), or S3226 (10^−5^ M) was perfused intraluminally for 30 minutes. When multiple doses of drugs were studied, they were perfused sequentially in the same animals. These doses were selected based on previously published data and similarities with clinical dosing. Forskolin (10^−4^ M) ± CFTR_inh_-172 (2 × 10^−5^ M) was perfused intraluminally for 45 minutes. After each experiment, the length of the duodenal test segment was measured in situ to the nearest 0.5 mm.

Sample volumes were measured by weight to the nearest 0.01 mg. The amount of bicarbonate in the effluents was quantitated by a validated micro back-titration method.^31^ Briefly, 100 μL of 5 × 10^−2^ M HCl was added to 2 mL sample with 2 mL of double-distilled water. Samples were then gassed with N_2_, prewashed in Ba(OH)_2_ to remove all CO_2_, and back-titrated with 2.5 × 10^−2^ M NaOH to an endpoint of pH 7.0 using an automated pH meter/titration unit (TIM 856 Radiometer, Copenhagen, Denmark). Bicarbonate secretion was determined in 15-min periods and expressed as micromoles per centimeter per hour and presented as bicarbonate output over time or net peak bicarbonate output (peak output minus average basal).

### In Vitro Measurement of Ion Transport and Bicarbonate Secretion.

For murine measurements, mice were anesthetized as above, and the proximal 1 cm of duodenum was removed and placed in ice-cold 3 × 10^−1^ M mannitol solution with indomethacin (10^−5^ M) to prevent endogenous prostaglandin formation. The mesentery was dissected off and the duodenum was opened along the mesenteric edge. The muscular layer was scraped from the mucosa using a glass slide while being bathed in the indomethacin-containing solution. Stripped duodenal mucosae were mounted between two 0.1 cm^2^ aperture sliders placed in temperature controlled Ussing chambers maintained at 37°C (P2300, Physiologic Instruments, San Diego, CA). For human endoscopic biopsy measurements, biopsies were collected in the same iso-osmolar mannitol solution with indomethacin and then mounted directly between two 0.031 cm^2^ aperture sliders. Tissue was bathed in apical solutions containing (in mM): NaCl 115, KGluconate 5.2, NaGluconate 25, MgCl_2_ 1.2, CaCl_2_ 1.2, Mannitol 10, gassed with 100% O_2_ and basolateral solutions containing (in mM): NaCl 115, K_2_HPO_4_ 2.4, KH_2_PO_4_ 0.4, NaHCO_3_ 25, MgCl_2_ 1.2, CaCl_2_ 1.2, Glucose 10, gassed with 95% O_2_/5% CO_2_. Ion transport was measured by short-circuit current (I_sc_), where tissues were voltage clamped at 0 mV using a voltage-clamp apparatus (VCC600, Physiologic Instruments, San Diego, CA). To monitor transepithelial resistance, the voltage clamp was released, and the open circuit voltage was recorded every 10 minutes. Resistance was calculated using Ohm’s Law. To measure bicarbonate secretion, we utilized the automatic pH-stat method, as previously described.^7,32^ In brief, a pH microelectrode was placed in the apical bath and set at the pH of the unbuffered apical solution (typically ~6.8). The titrator (Titrando 902, MetrOhm, Switzerland) was set to titrate 5 × 10^−3^ M HCl in 0.2 μL aliquots as the pH of the apical bath rose above the pH set point. Tiamo software (MetrOhm, Switzerland) was used to control the rate of titration and continuously measure the amount of HCl titrated into the apical bath and the apical bath pH. The set-up as described allows for simultaneous measurement of bicarbonate secretion and I_sc_ without electrical interference. Bicarbonate secretory rates (μmol/cm^2^•h) were calculated in 5-minute intervals by noting the amount titrated, the concentration of titrant, and the surface area of the slider aperture.

### Single cell sequencing analysis.

The R toolkit Seurat was employed to conduct an analysis and quality control of single-cell RNA sequencing (scRNA-seq) data from existing literature.^33^ We clustered the cells according to the Seurat default settings. In brief, we embedded the cells through a K-nearest neighbor graph, reduced the data with principal component analysis, and applied the Louvain algorithm for clustering. We visualized these clusters with UMAP (uniform manifold approximation and projection). To annotate major cell types and compare gene expression in crypts versus villi, we used the metadata annotations provided by Elmentaite et al.^19^ and Busslinger et al.,^18^ respectively. We utilized violin and feature plots to display gene expression profiles, and FeatureScatter to compare the co-expression of two genes by cell type.

### Human duodenal organoids.

Duodenal organoids were established and cultured as previously described.^34^ In short, crypt cells were isolated from duodenal endoscopic biopsies using a series of PBS washes followed by incubation with cold chelation buffer (distilled water with 5.6 mM Na_2_HPO_4_, 8.0 mM KH_2_PO_4_, 96.2 mM NaCl, 1.6 mM KCl, 43.4 mM sucrose, 54.9 mM d-sorbitol, 1 mM dl-dithiothreitol) with 2 μM EDTA for 30 mins on ice. After incubation, EDTA was removed, and the biopsies were vigorously resuspended in cold chelation buffer (without EDTA). The resuspension was allowed to sit on ice for 1 minute to allow biopsies to settle by gravity. The supernatant was collected. This process was repeated 3–5 times until no more crypts were present in the supernatant. The crypt containing supernatants were pooled and centrifuged at 600g for 5 minutes, followed by resuspension in 40 µL basement membrane extract /dome (BME, Culterex Pathclear Type 2 BME) and submerged in WENR complete media (ADMEM/F12, 1 mM HEPES, 1X Glutamax, 1 mM N-Acetylcysteine, 1X B-27, 0.5 µM A83–01, 1X Penicillin-Streptomycin-Glutamine, 10 mM Gastrin, 10 µM SB-202190, 50 ng/mL EGF, 10 mM Nicotinamide, 1X Normocin) with Rho-inhibitor (Y-27632 10 µM, Sigma Aldrich, St. Louis, MO) and CHIR99021 (10µM, Sigma Aldrich, St. Louis, MO) for first two days, followed by WENR complete media only until ready to passage. Duodenal enteroids were passaged by disrupting BME with cold (4°C) media, 1X cold (4°C) phosphate-buffer solution (PBS, without Ca^2+^ and Mg^2+^), then centrifuged at 600g for 3 minutes at 4°C. Supernatant was aspirated and single cells were isolated by adding warm (37°C) TrypLE Express (Gibco, Thermofisher Scientific, Waltham, MA) and incubated for 10 minutes at 37°C. Equal volume of fetal bovine serum was added to quench trypsin and cells were centrifuged at room temperature for 5 minutes at 600g. Pellet was resolubilized in BME (40 μL/dome) and plated in a 24-well tissue culture plate. Generation of apical-out enteroids was performed as previously described.^20^ Briefly, enteroids were dislodged from BME using with 1X cold (4°C) PBS (without Ca^2+^ and Mg^2+^) and resuspended in 5 mM EDTA in PBS for 1 hour at 4°C. Enteroids were then centrifuged at 200g for 3 minutes and resuspended in WENR or EN media (WENR without Wnt and R-Spondin) and seeded in ultra-low-attachment 24-well plates (Corning Costar, Thermofisher Scientific, Waltham, MA) for three days. On Day 3, undifferentiated and/or differentiated enteroids were harvested for experiments.

### Quantitative PCR.

Quantification of mRNA transcript levels in undifferentiated and differentiated apical-out enteroids was performed. Briefly, total RNA was extracted from fresh apical-out enteroids using Zymo Direct-Zol RNA miniprep kit (Zymo Research, Irvine, CA) according to the manufacturer’s instructions. Complementary DNA (cDNA) was synthesized from 1 to 2 μg of RNA using High-Capacity RNA-to-cDNA^™^ Kit (Zymo Research, Irvine, CA). Quantitative real-time polymerase chain reaction (qPCR) was performed on a StepOne Plus real-time PCR system (Applied Biosystems, Foster City, CA) using PowerUP SYBR Green Master Mix (Applied Biosystems, Foster City, CA). Each sample was run in triplicate in a 20 μL reaction volume containing 5 ng cDNA/reaction. PCR was carried out for 40 cycles at 95°C (denature) for 30 seconds, 60°C (anneal/extend) for 30 seconds and amplification data was collected after each cycle. The sequences of optimized gene-specific primers are listed in Supplemental Table 1. Glyceraldehyde-3-phosphate dehydrogenase (*GAPDH*) was used as the reference gene to obtain delta threshold cycles (∆Ct) values. The relative expression levels of the target genes were calculated using the 2^-∆∆Ct^ method.

### Confocal microscopy.

Treated Day 3 apical-out enteroids were fixed in 2% paraformaldehyde immediately after treatment and stored at 4°C until processing. All steps were performed in ultra-low attachment plates. Fixed enteroids were permeabilized for 30 minutes with permeabilization buffer (3% bovine serum albumin, BSA, ThermoFisher Scientific, Waltham, MA), 0.5% Triton X-100, and 0.7% Saponin (Sigma-Aldrich, St. Louis, MO) in PBS and blocked for 60 minutes with gentle shaking in blocking buffer (3% BSA, 0.2% Triton X-100). Samples were incubated overnight at 4°C with primary antibodies (Supplemental Table 2) diluted in antibody diluent (3% BSA, 0.2% Triton X-100, and 0.5% Saponin in PBS), washed 4 times, 30 minutes each in antibody diluent buffer, and incubated with secondary antibodies (Table 2) for 1 hour with gentle shaking at room temperature. Samples were washed 4 times for 30 minutes each in antibody diluent buffer. Samples were then carefully resuspended in SlowFade gold antifade mounting medium (ThermoFisher Scientific, Waltham, MA), mounted on microscope slides and sealed with glass coverslips (1.0 mm) (ThermoFisher Scientific, Waltham, MA). Slides were imaged using Leica Stellaris 8 Confocal microscope. Z-stacks of 0.33 μm sections/sample were obtained and analyzed using FIJI Software (ImageJ). Following acquisition, image settings were adjusted to optimize the signal to noise ratio. For quantitative analysis, the same settings were used across the different conditions compared, and the protein-of-interest mean fluorescence intensity (MFI) after background subtractions was analyzed. Villin was used as a marker for the plasma membrane and used to identify the area for fluorescence intensity measurements. Drug-treated MFI measurements were normalized to corresponding vehicle control MFI measurements.

### Statistics.

Results are expressed as means ± standard error of the mean (SEM) for a series of n experiments. Statistical analysis was performed by using the Student’s t-test for unpaired data or by ANOVA with the Student-Newman Keuls post hoc test, as appropriate. P values of <0.05 were considered significant.

### Study Approval.

C57BL/6J mice from Jackson Laboratories (Bar Harbor, ME) were used as wildtype control mice. CFTR^tm1Unc^ knockout (KO) mice were obtained from Case Western University CF Mouse Models Core Facility and maintained on 50% PEG-3350 with electrolytes solution in their drinking water with standard chow. All animal protocols were approved by the Institutional Animal Care and Use Committee at Stanford University (#33183). Children and adults undergoing upper endoscopy for clinical indications were approached to obtain additional duodenal biopsies for research under an Institutional Review Board approved protocol (#47536). Subjects without endoscopic and microscopic evidence of duodenal pathology were included in this study.

## Supplementary Material

Supplement 1

## References

[1] Youngberg C. A. Comparison of gastrointestinal pH in cystic fibrosis and healthy subjects. Digestive diseases and sciences 32, 472–480 (1987). 3646103 10.1007/BF01296029

[2] Gustafsson J. K. Bicarbonate and functional CFTR channel are required for proper mucin secretion and link cystic fibrosis with its mucus phenotype. The Journal of experimental medicine 209, 1263–1272 (2012). https://doi.org:10.1084/jem.20120562 22711878 PMC3405509

[3] Seidler U. E. Gastrointestinal HCO3- transport and epithelial protection in the gut: new techniques, transport pathways and regulatory pathways. Curr Opin Pharmacol 13, 900–908 (2013). https://doi.org:10.1016/j.coph.2013.10.001 24280619

[4] Busby R. W. Linaclotide, through activation of guanylate cyclase C, acts locally in the gastrointestinal tract to elicit enhanced intestinal secretion and transit. Eur J Pharmacol 649, 328–335 (2010). https://doi.org:10.1016/j.ejphar.2010.09.019 20863829

[5] Ahsan M. K. Linaclotide activates guanylate cyclase-C/cGMP/protein kinase-II-dependent trafficking of CFTR in the intestine. Physiol Rep 5 (2017). https://doi.org:10.14814/phy2.13299 PMC5471438 28592587

[6] McHugh D. R. Linaclotide improves gastrointestinal transit in cystic fibrosis mice by inhibiting sodium/hydrogen exchanger 3. Am J Physiol Gastrointest Liver Physiol 315, G868–G878 (2018). https://doi.org:10.1152/ajpgi.00261.2017 30118317 PMC9925117

[7] Sellers Z. M. Heat-stable enterotoxin of Escherichia coli stimulates a non-CFTR-mediated duodenal bicarbonate secretory pathway. Am J Physiol Gastrointest Liver Physiol 288, G654–663 (2005). https://doi.org:10.1152/ajpgi.00386.2004 15513951

[8] Sellers Z. M. Heat-stable enterotoxin of Escherichia coli (STa) can stimulate duodenal HCO3(−) secretion via a novel GC-C- and CFTR-independent pathway. FASEB journal : official publication of the Federation of American Societies for Experimental Biology 22, 1306–1316 (2008). https://doi.org:10.1096/fj.06-7540com 18096816

[9] Tan Q. Inhibition of Na(+) /H(+) exchanger isoform 3 improves gut fluidity and alkalinity in cystic fibrosis transmembrane conductance regulator-deficient and F508del mutant mice. Br J Pharmacol 178, 1018–1036 (2021). https://doi.org:10.1111/bph.15323 33179259

[10] Arora K. Guanylate cyclase 2C agonism corrects CFTR mutants. JCI Insight 2 (2017). https://doi.org:10.1172/jci.insight.93686 PMC5841874 28978796

[11] Pratha V. S. Identification of transport abnormalities in duodenal mucosa and duodenal enterocytes from patients with cystic fibrosis. Gastroenterology 118, 1051–1060 (2000). 10833480 10.1016/s0016-5085(00)70358-1

[12] Ma T. Thiazolidinone CFTR inhibitor identified by high-throughput screening blocks cholera toxin-induced intestinal fluid secretion. J Clin Invest 110, 1651–1658 (2002). https://doi.org:10.1172/JCI16112 12464670 PMC151633

[13] Snouwaert J. N. An animal model for cystic fibrosis made by gene targeting. Science 257, 1083–1088 (1992). https://doi.org:10.1126/science.257.5073.1083 1380723

[14] Lee S., Cil O., Haggie P. M. & Verkman A. S. 4,8-Dimethylcoumarin Inhibitors of Intestinal Anion Exchanger slc26a3 (Downregulated in Adenoma) for Anti-Absorptive Therapy of Constipation. J Med Chem 62, 8330–8337 (2019). https://doi.org:10.1021/acs.jmedchem.9b01192 31389695 PMC7034394

[15] Wang Z., Petrovic S., Mann E. & Soleimani M. Identification of an apical Cl(−)/HCO3(−) exchanger in the small intestine. Am J Physiol Gastrointest Liver Physiol 282, G573–579 (2002). https://doi.org:10.1152/ajpgi.00338.2001 11842009

[16] Foulke-Abel J. Human Enteroids as a Model of Upper Small Intestinal Ion Transport Physiology and Pathophysiology. Gastroenterology 150, 638–649 e638 (2016). https://doi.org:10.1053/j.gastro.2015.11.047 26677983 PMC4766025

[17] Yin J. Molecular Basis and Differentiation-Associated Alterations of Anion Secretion in Human Duodenal Enteroid Monolayers. Cellular and Molecular Gastroenterology and Hepatology 5, 591–609 (2018). 29930980 10.1016/j.jcmgh.2018.02.002 PMC6009799

[18] Busslinger G. A. Human gastrointestinal epithelia of the esophagus, stomach, and duodenum resolved at single-cell resolution. Cell Rep 34, 108819 (2021). https://doi.org:10.1016/j.celrep.2021.108819 33691112

[19] Elmentaite R. Cells of the human intestinal tract mapped across space and time. Nature 597, 250–255 (2021). https://doi.org:10.1038/s41586-021-03852-1 34497389 PMC8426186

[20] Co J. Y. Controlling Epithelial Polarity: A Human Enteroid Model for Host-Pathogen Interactions. Cell Rep 26, 2509–2520 e2504 (2019). https://doi.org:10.1016/j.celrep.2019.01.108 30811997 PMC6391775

[21] Chen T. Myosin VI mediates the movement of NHE3 down the microvillus in intestinal epithelial cells. J Cell Sci 127, 3535–3545 (2014). https://doi.org:10.1242/jcs.149930 24928903 PMC4132392

[22] Wen G. Helicobacter pylori infection downregulates duodenal CFTR and SLC26A6 expressions through TGFbeta signaling pathway. BMC Microbiol 18, 87 (2018). https://doi.org:10.1186/s12866-018-1230-8 30119655 PMC6098588

[23] Gelfond D. Impact of CFTR Modulation on Intestinal pH, Motility, and Clinical Outcomes in Patients With Cystic Fibrosis and the G551D Mutation. Clin Transl Gastroenterol 8, e81 (2017). https://doi.org:10.1038/ctg.2017.10 28300821 PMC5387753

[24] Braga Emidio N. Improving the Gastrointestinal Stability of Linaclotide. J Med Chem 64, 8384–8390 (2021). https://doi.org:10.1021/acs.jmedchem.1c00380 33979161 PMC8237258

[25] Rao S. P. A role for guanylate cyclase C in acid-stimulated duodenal mucosal bicarbonate secretion. Am J Physiol Gastrointest Liver Physiol 286, G95–G101 (2004). https://doi.org:10.1152/ajpgi.00087.2003 12881226

[26] Strubberg A. M. Cftr Modulates Wnt/beta-Catenin Signaling and Stem Cell Proliferation in Murine Intestine. Cell Mol Gastroenterol Hepatol 5, 253–271 (2018). https://doi.org:10.1016/j.jcmgh.2017.11.013 29675451 PMC5904038

[27] Kaunitz J. D. & Akiba Y. Duodenal intracellular bicarbonate and the ‘CF paradox’. JOP 2, 268–273 (2001). 11875270

[28] Bijvelds M. J. C. Rescue of chloride and bicarbonate transport by elexacaftor-ivacaftor-tezacaftor in organoid-derived CF intestinal and cholangiocyte monolayers. J Cyst Fibros (2021). https://doi.org:10.1016/j.jcf.2021.12.006 34922851

[29] Walker N. M. Role of down-regulated in adenoma anion exchanger in HCO3-secretion across murine duodenum. Gastroenterology 136, 893–901 (2009). https://doi.org:10.1053/j.gastro.2008.11.016 19121635 PMC2694732

[30] Singh A. K. Molecular transport machinery involved in orchestrating luminal acid-induced duodenal bicarbonate secretion in vivo. J Physiol 591, 5377–5391 (2013). https://doi.org:10.1113/jphysiol.2013.254854 24018950 PMC3936374

[31] Hogan D. L. CFTR mediates cAMP- and Ca2+-activated duodenal epithelial HCO3- secretion. Am J Physiol 272, G872–878 (1997). https://doi.org:10.1152/ajpgi.1997.272.4.G872 9142920

[32] Pratha V. S. Utility of endoscopic biopsy samples to quantitate human duodenal ion transport. The Journal of laboratory and clinical medicine 132, 512–518 (1998). 9851742 10.1016/s0022-2143(98)90130-5

[33] Hao Y. Integrated analysis of multimodal single-cell data. Cell 184, 3573–3587 e3529 (2021). https://doi.org:10.1016/j.cell.2021.04.048 34062119 PMC8238499

[34] Sato T. Long-term expansion of epithelial organoids from human colon, adenoma, adenocarcinoma, and Barrett’s epithelium. Gastroenterology 141, 1762–1772 (2011). https://doi.org:10.1053/j.gastro.2011.07.050 21889923

