# Supplementary material for: Critical role of down-regulated in adenoma bicarbonate transporter in linaclotide stimulated intestinal bicarbonate secretion
Source: bioRxiv. 2023 May 7:2023.05.05.539132. Preprint. [Version 1] doi: 10.1101/2023.05.05.539132 (PMC10187319; doi:10.1101/2023.05.05.539132)
Supplement: Supplementary file 1 [file media-1.pdf]

SUPPLEMENTARY TABLES AND FIGURES

Supplementary Table 1. qPCR gene-specific Primer sequences

Gene	Forward Primer (5′-3′)	Reverse Primer (5′-3′)
SLC26A3	CAGCCCCCTATTACACCTGA	CCTCCTGTGCTCTCCTGAAC
SLC26A6	GCCTTGAACGACTCCATGAT	TGTGAGACGAAGACCTGCAC
CFTR	CCTATGACCCGGATAACAAGGA	GAACACGGCTTGACAGCTTTA
SLC9A3	CCTGACCATCAAGCCTCTGG	ACATTCAGGATCCGGTCTCG
LGR5	GATGTTGCTCAGGGTGGACT	GGGAGCAGCTGACTGATGTT
GUCY2C	GGCTGTCCTTTAGTTCCCAGG	GAAAGTAGCGTTCACAGTCACAT
MYO6	TAACCCACTCCTAGAAGCCTTT	GCACCAGCACACAACCTATAA
GAPDH	GAAGGTGAAGGTCGGAGTC	GAAGATGGTGATGGGATTTC
SLC26A3 (down-regulated in adenoma, DRA), SLC26A6 (putative anion transporter-1, PAT-1), CFTR (cystic fibrosis transmembrane conductance regulator), SLC9A3 (sodium/hydrogen exchanger 3, NHE3), LGR5 (leucine rich repeat containing G protein-coupled receptor 5), GUCY2C (guanylate cyclase 2C), MYO6 (myosin VI), GAPDH (glyceraldehyde-3-phosphate dehydrogenase)		

Supplementary Table 2. List of antibodies

Primary Antibodies	Dilution	Manufacturer
**DRA (mouse)**	1:200	Santa Cruz (sc-376187)
**NHE3 (rabbit)**	1:400	Novus Biologicals (NBP1-82574)
**Villin (rabbit)**	1:400	ThermoFisher (PA5-29078)
**Villin (mouse) 1D2C3**	1:200	Santa Cruz (sc-58897)
**Hoechst 33342**	Per manufacturer's protocol	Abcam (ab228551)

Secondary antibodies	Dilution	Manufacturer
**Goat Anti-Rabbit Alexa flour 594**	1:300	Abcam (ab150088)
**Goat Anti-Mouse Alexa flour 647**	1:300	Abcam (ab150115)
**Goat Anti-Mouse IgG H&L (Alexa Fluor® 488)**	1:1000	Abcam (ab150113)

SUPPLEMENTARY FIGURES

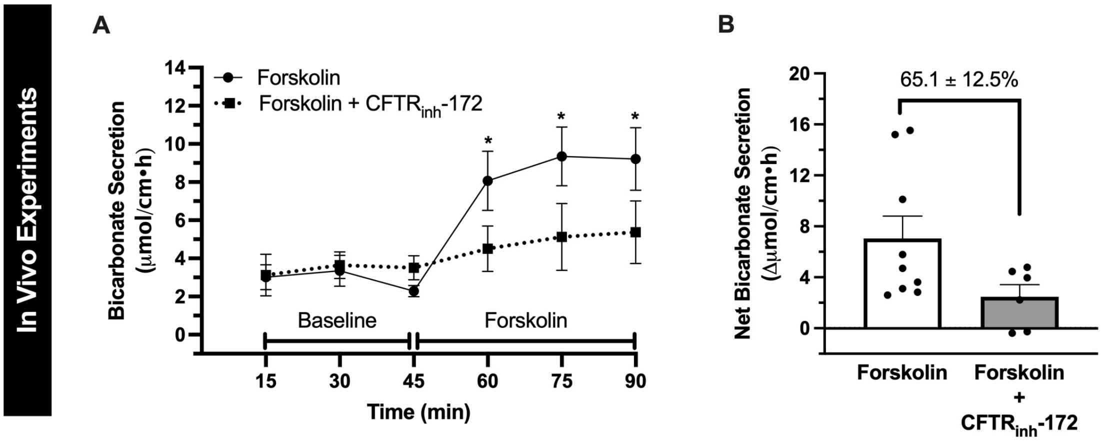

Supplemental Figure 1. $CFTR_{inh}$-172 inhibits forskolin-stimulated duodenal bicarbonate secretion in mice. A. To compare the effect of pharmacologic CFTR inhibition with CFTRinh-172 on linaclotide and forskolin-stimulated bicarbonate secretion, we performed similar in vivo experiments as Fig. 2a. Duodenum of wildtype mice were perfused with saline then forskolin (10^{-4} M) (black circles) or saline + $CFTR_{inh}$-172 (2×10^{-5} M) then forskolin (10^{-4} M) (black squares). Bicarbonate secretion was calculated from the perfusates similar to Fig. 2a. *, $P<0.05$ by ANOVA. **B.** Comparison of net change in forskolin-stimulated bicarbonate secretion (peak response – baseline secretion) in the presence or absence of $CFTR_{inh}$-172. Columns with whiskers represent mean ± SEM with each dot representing a different experiment. Mean ± SEM percent inhibition indicated above columns.

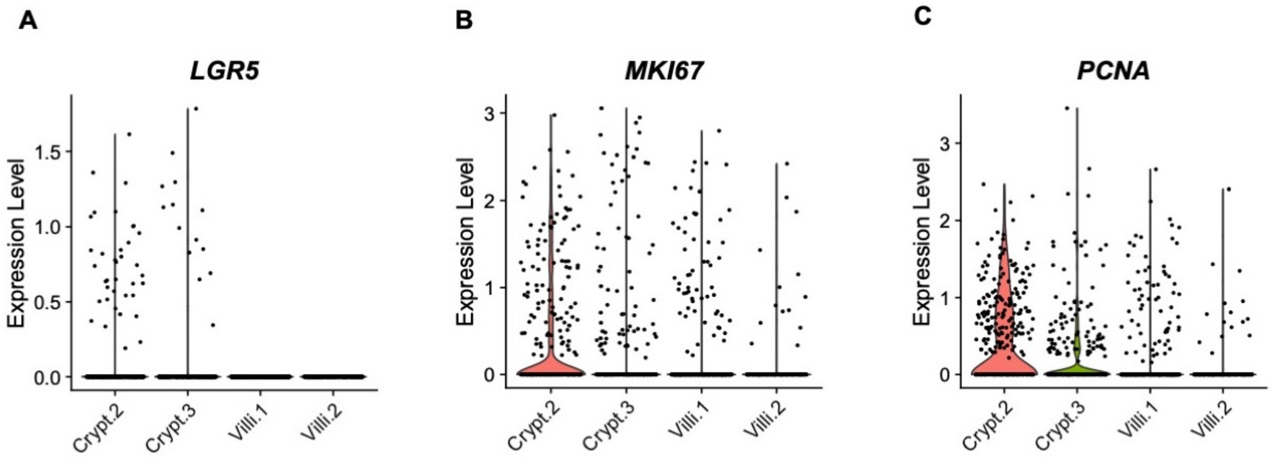

Supplementary Figure 2. Validation of previously published human duodenum scRNA-seq datasets. A-C. To verify the crypt vs. villi identity in the Busslinger et al. dataset we examined the expression of *LGR5* **(A)**, *MKI67* **(B)**, and *PCNA* **(C)**.

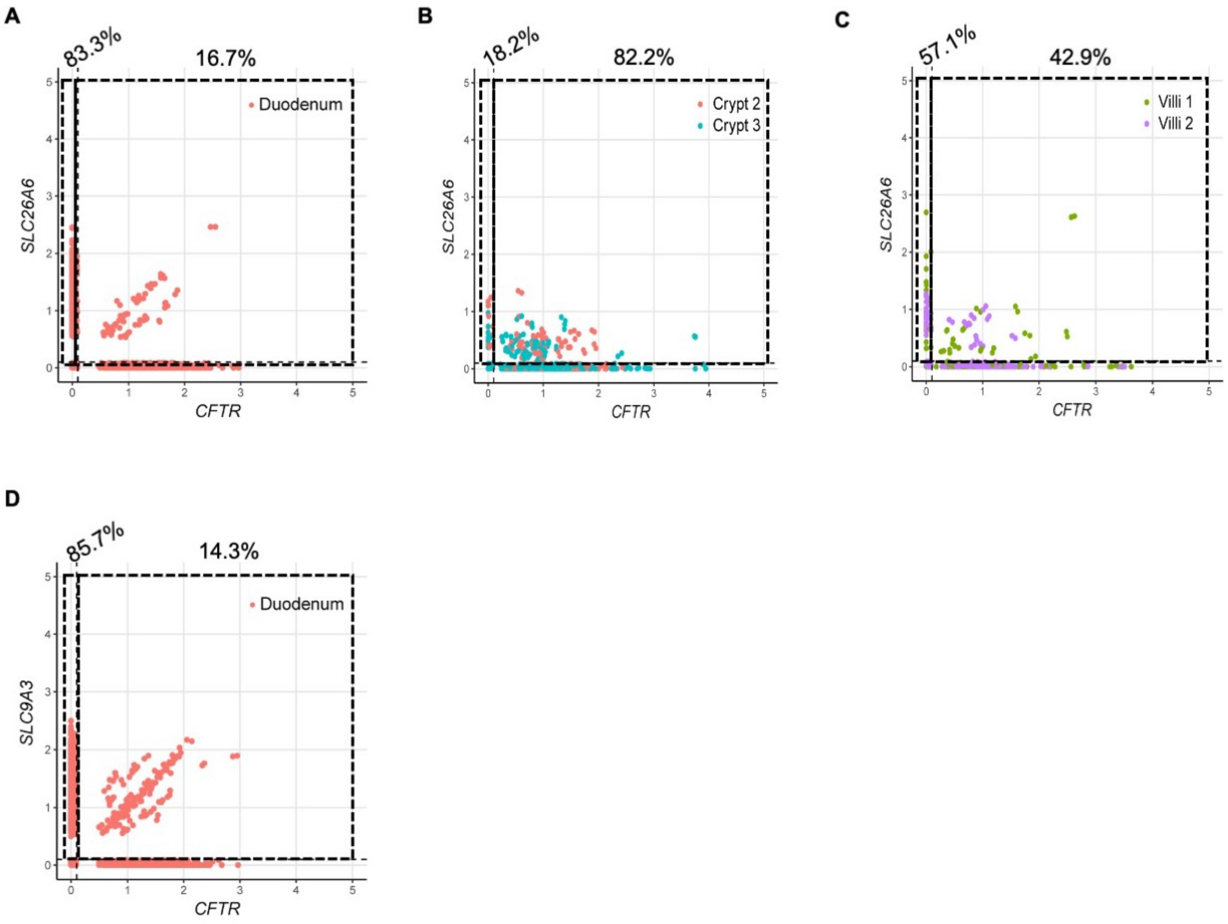

Supplementary Figure 3. Co-expression of *CFTR* and *SLC26A6* (PAT-1) and *SLC9A3* (NHE3) from human duodenal sc-RNAseq data. A-C. Co-expression of *SLC26A6* (PAT-1) and *CFTR* mRNA using FeatureScatter based on Elmentaite et al.[14] enterocytes (A) and Busslinger et al.[15] crypt and villi (B and C) datasets. **D.** Co-expression of *SLC9A3* (NHE3) and *CFTR* mRNA using FeatureScatter based on Elmentaite et al. enterocytes. There were insufficient numbers of *SLC9A3* positive cells in the Busslinger et al. dataset to do the same analysis as B and C. Numbers on top of graphs represent the percentage of *SLC26A6*- or *SLC9A3*-expressing cells that express these only (left) or *SLC26A6/SLC9A3* and *CFTR* (right).

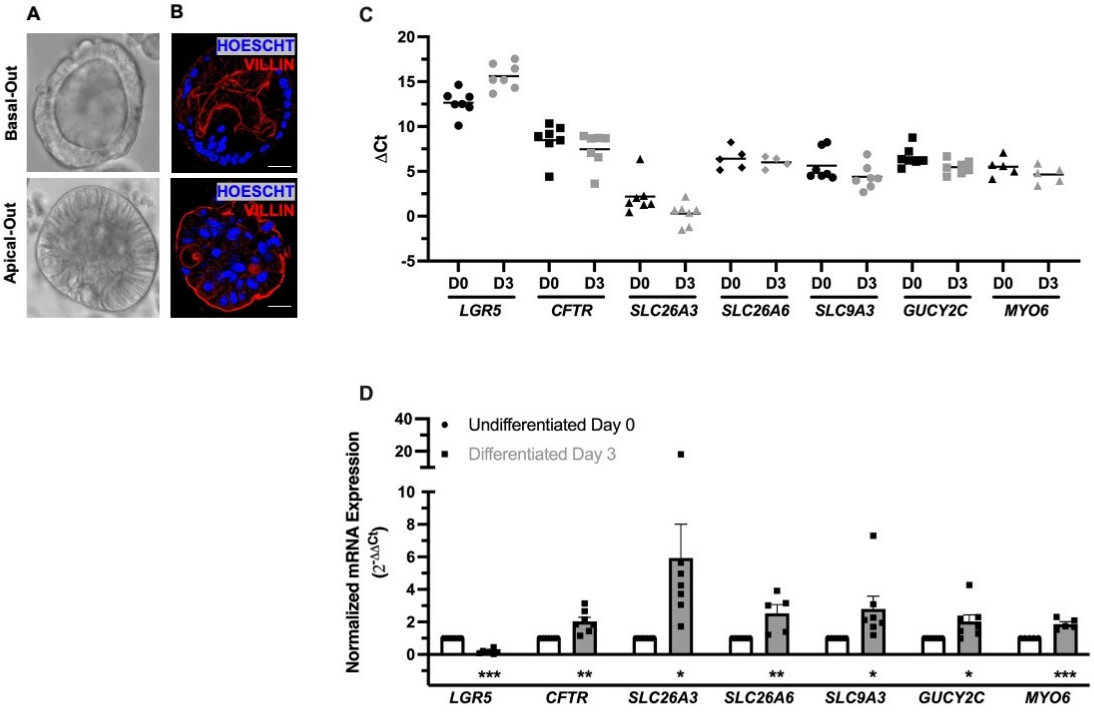

Supplementary Figure 4. Characterization of Apical-Out Duodenal Enteroids. A. Sample brightfield microscope images of basal-out (top) and apical-out (bottom) human duodenal enteroids. **B.** Representative confocal immunofluorescent images of basal-out (top) and apical-out (bottom) human duodenal enteroids stained with villin (apical membrane) and Hoescht (cell nucleus). Scale bar = 20 μm. **C and D.** mRNA expression of *LGR5, CFTR, SLC26A3, SLC26A6, SLC9A3, GUCY2C, and MYO6* in undifferentiated and differentiated (x3 days) apical-out human duodenal enteroids. Delta CT was calculated by comparing to *GAPDH* expression (C) and Delta Delta CT was calculated by comparing Differentiated Day 3 enteroids to Undifferentiated Day 0 enteroids. *, $P<0.05$; **, $P<0.01$; ***, $P<0.001$ compared to Undifferentiated Day 0 enteroids by unpaired Student's t-test.

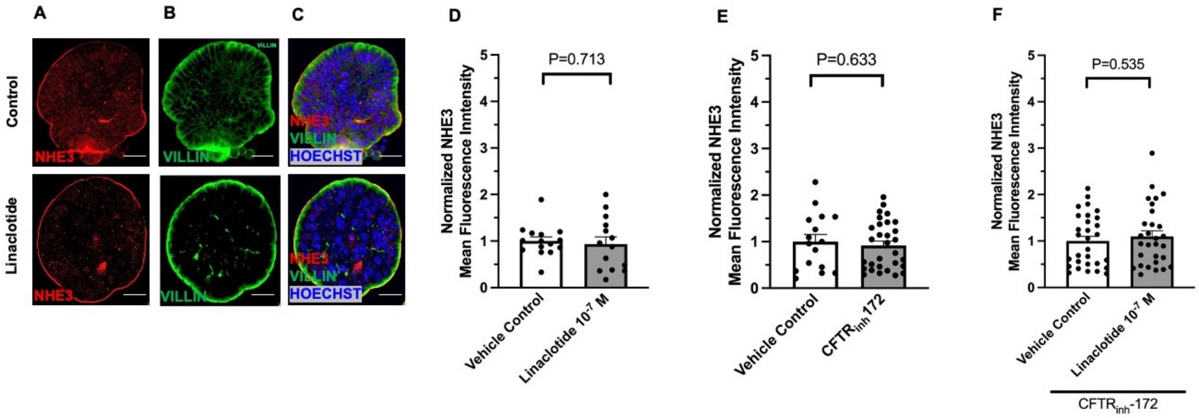

Supplemental Figure 5. Linaclotide does not change the membrane expression of NHE3 in apical-out human duodenal enteroids. A-C. Representative confocal microscopy images of NHE3 (A), villin (B), and NHE3 and villin and Hoescht (C) during control conditions (water, 40 minutes, top) or linaclotide (10^{-7} M, 40 minutes, bottom). **D-F.** Quantification of NHE3 present at the apical membrane using villin to define apical membrane, similar to Fig. 6. Apical membrane NHE3 mean fluorescence intensity following $CFTR_{inh}$-172 (2×10^{-5} M, 40 minutes) and/or linaclotide (10^{-7} M, 40 minutes) treatment was normalized to vehicle controls for comparison. Vehicle control for linaclotide was water and for $CFTR_{inh}$-172 was DMSO. Columns with whiskers are mean $\pm$ SEM with each dot representing a different enteroid. Enteroids from three different patients were used for each condition. Significance determined by unpaired Student's t-test. For enteroids treated with both $CFTR_{inh}$-172 and linaclotide, enteroids were pretreated with $CFTR_{inh}$-172 for 40 minutes prior to linaclotide treatment.
